# Haptoglobin treatment contributes to regulating nitric oxide signal and reduces oxidative stress in the penis: A preventive treatment for priapism in sickle cell disease

**DOI:** 10.3389/fphys.2022.961534

**Published:** 2022-09-13

**Authors:** Pamela da Silva Pereira, Dalila Andrade Pereira, Fabiano Beraldi Calmasini, Leonardo O. Reis, Nathan Brinkman, Arthur L. Burnett, Fernando Ferreira Costa, Fábio Henrique Silva

**Affiliations:** ^1^ Hematology and Hemotherapy Center, University of Campinas, Campinas, Brazil; ^2^ Laboratory of Multidisciplinary Research, São Francisco University Medical School, Bragança Paulista, Brazil; ^3^ UroScience, Pontifical Catholic University of Campinas, PUC-Campinas, Campinas, Brazil; ^4^ CSL Behring LLC, Kankakee, IL, United States; ^5^ The James Buchanan Brady Urological Institute and Department of Urology, The Johns Hopkins School of Medicine, Baltimore, MD, United States

**Keywords:** intravascular hemolysis, PDE5, NADPH oxidase, cGMP, erectile dysfunction

## Abstract

**Background:** In sickle cell disease (SCD), reduced bioavailability of endothelial NO and cGMP results in reduced expression of phosphodiesterase type 5 (PDE5), thus impairing the penile erection control mechanism and resulting in prolonged penile erection (priapism). In SCD, reduced NO bioavailability is associated with excess plasma hemoglobin due to intravascular hemolysis and increased oxidative stress. Haptoglobin is the plasma protein responsible for reducing plasma hemoglobin levels, but in SCD, haptoglobin levels are reduced, which favors the accumulation of hemoglobin in plasma. Therefore, we aimed to evaluate the effects of haptoglobin treatment on functional and molecular alterations of erectile function, focusing on the contractile and relaxant mechanisms of corpus cavernosum (CC), as well as oxidative stress.

**Methods:** SCD mice were treated with haptoglobin (400 mg/kg, subcutaneous) or vehicle of Monday, Wednesday and Friday for a period of 1 month. Corpus cavernosum strips were dissected free and placed in organ baths. Cumulative concentration-response curves to the acetylcholine, sodium nitroprusside, phenylephrine and KCL, as well as to electrical field stimulation (EFS), were obtained in CC. Protein expressions of eNOS, phosphorylation of eNOS at Ser-1177, nNOS, PDE5, ROCK1, ROCK2, gp91^phox^, 3-nitrotyrosine, and 4-HNE were measured by western blot in CC.

**Results:** Increased CC relaxant responses to acetylcholine, sodium nitroprusside and electrical-field stimulation were reduced by haptoglobin in SCD mice. Reduced CC contractile responses to phenylephrine and KCl were increased by haptoglobin in SCD mice. Haptoglobin prevented downregulated eNOS, p-eNOS (Ser-1177), PDE5, and ROCK2 protein expressions and reduced protein expressions of reactive oxygen species markers, NADPH oxidase subunit gp91phox, 3-nitrotyrosine and 4-HNE in penises from SCD mice. Haptoglobin treatment did not affect ROCK1 and nNOS protein expressions in penises from SCD mice. Basal cGMP production was lower in the SCD group, which was normalized by haptoglobin treatment.

**Conclusion:** Treatment with haptoglobin improved erectile function due to up-regulation of eNOS-PDE5 expression and down-regulation of the gp91phox subunit of NADPH oxidase and oxidative/nitrosative stress in the penises of SCD mice. Treatment with haptoglobin also increased contractile activity due to up-regulation of ROCK2. Therefore, haptoglobin treatment may be an additional strategy to prevent priapism in SCD.

## Introduction

A point mutation in the β-globin gene causes sickle cell disease (SCD), the most common single-gene disease. This alteration promotes the polymerization of hemoglobin under conditions of reduced oxygenation, causing rigid sickle-shaped red blood cells and hemolytic anemia. SCD affects millions of people worldwide, approximately 100,000 Americans, and is most common in sub-Saharan Africa (Telen et al., 2019). Priapism is characterized by prolonged and painful penile erection in the absence of sexual stimulation or excitement (Salonia et al., 2014). Ischemic priapism is prevalent in men with SCD; it is caused by slight or absent intracorporal blood flow and is characterized by painful erections because of blood stagnation within CC (veno-occlusion) (Ericson et al., 2021). Recurrent events of priapism promote fibrosis in the erectile tissue, which results in permanent erectile dysfunction (Musicki and Burnett, 2020). Clinical studies have shown that 30–45% of men with SCD have episodes of ischemic priapism and of these 30% progress to erectile dysfunction (Mantadakis et al., 1999; Adeyoju et al., 2002; Alvaia et al., 2020). Despite the high incidence of priapism, the main treatments available do not prevent priapism (Salonia et al., 2014).

In SCD, the reduction in NO bioavailability is associated with intravascular hemolysis and increased oxidative stress (Gladwin and Sachdev, 2012). Under pathological conditions, NADPH oxidase (NOX) is the primary precursor for the formation of large amounts of intravascular superoxide anion. In the penis of SCD mice, the isoform of NADPH oxidase-2 (gp91phox, NOX2) is one of the primary sources responsible for increased oxidative stress, along with the uncoupled eNOS and xanthine oxidase (Bivalacqua et al., 2013; Silva et al., 2016b; Musicki et al., 2018). The reduced bioavailability of NO and increased oxidative stress in the penis have been identified as one of the main causes of triggering priapism in men and mice with SCD (Champion et al., 2005; Kato, 2012; Lagoda et al., 2013; Silva et al., 2016b). Experimental evidence suggests that reduced NO/cGMP bioavailability results in a compensatory reduction in PDE5 activity and expression in the CC, thus impairing the mechanism that controls excessive penile erection (Champion et al., 2005; Bivalacqua et al., 2012; Silva et al., 2016b). Furthermore, men and mice with SCD show lower expression and activity of the RhoA-ROCK pathway, thus impairing the maintenance of the flaccid state of the penis and contributing to priapism (Bivalacqua et al., 2010; Lagoda et al., 2013).

The process of intravascular hemolysis leads to the release of hemoglobin into the plasma (Reiter et al., 2002). Under physiological conditions, haptoglobin is the plasma protein responsible for defending the body against the accumulation of free hemoglobin (Buehler et al., 2020). In plasma, haptoglobin binds to free hemoglobin forming a hemoglobin-haptoglobin complex that is metabolized by macrophages in the reticuloendothelial system. Macrophages express the CD163 receptor that mediates endocytosis and internalization of the hemoglobin-haptoglobin complex (Buehler et al., 2020). However, in SCD, high concentrations of hemoglobin are released into the plasma, depleting haptoglobin and thus accumulating free hemoglobin in the plasma (Muller-Eberhard et al., 1968; Reiter et al., 2002; Santiago et al., 2018). Hemoglobin in plasma or interstitial space reacts with NO, generating nitrate and methemoglobin (Reiter et al., 2002). The inactivation of NO by plasma hemoglobin may probably contribute to priapism in SCD. In fact, clinical studies point to a strong positive correlation between priapism and elevated levels of intravascular hemolysis in men with SCD (Nolan et al., 2005; Kato et al., 2006; Cita et al., 2016). Recent studies have reported that haptoglobin treatment inhibits vaso-occlusion in SCD mice and preserves vascular NO during hemolysis (Schaer et al., 2016; Shi et al., 2016; Belcher et al., 2018). Therefore, we hypothesized that haptoglobin treatment may reverse the exaggerated erectile response induced by stimulation of the NO-cGMP pathway by normalizing eNOS and PDE5 expression in the penis of SCD mouse, as well as normalizing the contractile activity of CC and increased oxidative stress.

## Materials and methods

### Ethical approval

All protocols in this study were approved by the Committee for Ethics in Animal Experimentation of the University of Campinas (IACUC/CEEA-UNICAMP, Permit number 4754-1/2017).

### Animals and treatment

Animal procedures and experimental protocols were performed in accordance to the Ethical Principles in Animal Research adopted by the Brazilian College for Animal Experimentation and followed the Guide for the Care and Use of Laboratory Animals. All mice strains were originally purchased from Jackson Laboratories (Bar Harbor, ME). Characterization and breeding were performed at the Multidisciplinary Center for the Investigation of Biological Science in Laboratory Animals of the University of Campinas (CEMIB). We used C57BL/6 male mice (wild-type, WT) and Berkeley transgenic SCD mice, aged 3–4 months old, housed three per cage on a 12 h light-dark cycle. SCD male mice were treated with haptoglobin (400 mg/kg, subcutaneous) or vehicle every of Monday, Wednesday, and Friday for a period of 1 month (Shi et al., 2016). Transgenic SCD mice express exclusively human sickle hemoglobin (HbS), generated by knockout of mouse alpha and beta globins and insertion of a single transgene that expresses human alpha and beta S globin. SCD mice are obtained by interbreeding sickle cell males with hemizygous females in-house (Pászty et al., 1997). Genotyping was performed by CEMIB (Campinas, SP, BR).

### Functional studies in cavernosal strips and concentration-response curves

Strips of mouse CC were mounted in a 7-ml organ system containing Krebs solution at 37°C, continuously bubbled with a mixture of 95% O_2_ and 5% CO_2_ (pH 7.4) and suspended between two metal hooks. One hook was connected to a force transducer and the other acted as a fixed attachment point. Tissues were allowed to equilibrate for 60 min under a resting tension of 2.5 mn. The isometric force was recorded using a PowerLab 400™ data acquisition system (Software LabChart, version 7.0, AD Instrument, MA, United States). Cumulative concentration-response curves were constructed for the muscarinic agonist acetylcholine (ACh; 10^–9^ to 10^–5^ M) and sodium nitroprusside (SNP; 10^–8^ to 10^–4^ M) in cavernosal strips pre-contracted with phenylephrine (10^–5^ M). Cumulative concentration–response curves to the contractile agent phenylephrine (α1-adrenergic receptor agonist, 10^–8^ to 3 × 10^–4^ M) and KCl (3 × 10^–4^ to 3 × 10^–1^ M) were obtained in cavernosal strips. Nonlinear regression analysis to determine the pEC_50_ was carried out using GraphPad Prism (GraphPad Software, San Diego, CA, United States).

### Electrical-field stimulation (EFS)

EFS was applied on cavernosal strips placed between two platinum ring electrodes connected to a Grass S88 stimulator (Astro-Med Industrial Park, RI, United States). EFS was conducted at 50 V, 1 m pulse width and trains of stimuli lasting 10 s at varying frequencies. In order to study the nitrergic cavernosal relaxations, tissues were pretreated with guanethidine (3 × 10^–5^ M; to deplete the catecholamine stores of adrenergic fibers) and atropine (10^–6^ M; to produce muscarinic antagonism) for 30 min prior to pre-contraction with phenylephrine (10^–5^ M). When a stable contraction level was attained, a series of EFS-induced relaxations were constructed (2–32 Hz).

### Western blot analysis

CC tissue was homogenized in lysis buffer and centrifuged at 12,000 g for 20 min at 4°C. Homogenates containing 50 μg total proteins were run on 4–20% Tris-HCl gels (Bio-Rad Laboratories, Hercules, CA, United States) and transferred to a nitrocellulose membrane. Nonfat dry milk 5% (Bio-Rad) in Tris-buffered saline/Tween was used for 1 h at 24°C to block nonspecific binding sites. Membranes were incubated for 15–16 h at 4°C with the following antibodies: monoclonal anti-3-NT (1:1,000, Abcam, catalog number ab7048, Cambridge, MA), polyclonal anti-4-HNE antibody (1:1,000, catalog number ab46545, Abcam), monoclocal anti-p-eNOS (Ser-1177) antibody (1:1,000, Abcam, catalog number ab215717), polyclonal anti-eNOS antibody (1:1,000, Abcam, catalog number ab5589), polyclonal anti-nNOS antibody (1:1,000, Abcam catalog number ab5586), polyclonal anti-PDE5 (1:500; catalog number ab64179, Abcam), monoclonal anti-ROCK-1/ROCK-2 (1:1,000; catalog number ab134181/ab125025 Abcam), gp91phox (1:1,000; BD Transduction Laboratories, catalog number 611414, San Diego, CA) and monoclonal anti-β-actin (1:7,000; Sigma-Aldrich, catalog number A5316 St. Louis, MO). Densitometry was analyzed using the ImageJ Software (National Institute of Health, Bethesda-MD, United States). Quantified densitometry results were normalized to β-actin. Quantified densitometry results of eNOS phosphorylated at Ser1177 were normalized to total eNOS.

### Determination of cGMP levels

Quantitative assays for cGMP were performed using a commercial enzyme immunoassay kit (Cayman Chemical Cyclic GMP EIA kit, Ann Arbor, MI, United States). For penile cGMP content, frozen penile tissue was homogenized in 5% trichloroacetic acid and centrifuged. TCA was extracted from the supernatant with three washes of water-saturated ether. cGMP was expressed as pmol/mg tissue.

### Drugs

ACh, phenylephrine, guanethidine and, atropine were acquired from Sigma-Aldrich (St Louis, MO, United States). The human haptoglobin (Hp) solution was a kind gift from CSL Behring (Kankakee, IL, United States). The analytical grade was required for all reagents. Either deionized water was used as solvents, and working solutions were diluted prior to use.

### Statistical analysis

The GraphPad Prism Program (GraphPad Software Inc.) was used for statistical analysis. Data are expressed as the mean ± SEM of N experiments. Statistical comparisons were made using one-way analysis of variance (ANOVA), and tukey method was chosen as a post-test. A value of *p* < 0.05 was considered statistically significant.

### Haptoglobin treatment corrects exaggerated corpus cavernosum relaxations in sickle cell disease mice

**FIGURE 1:**
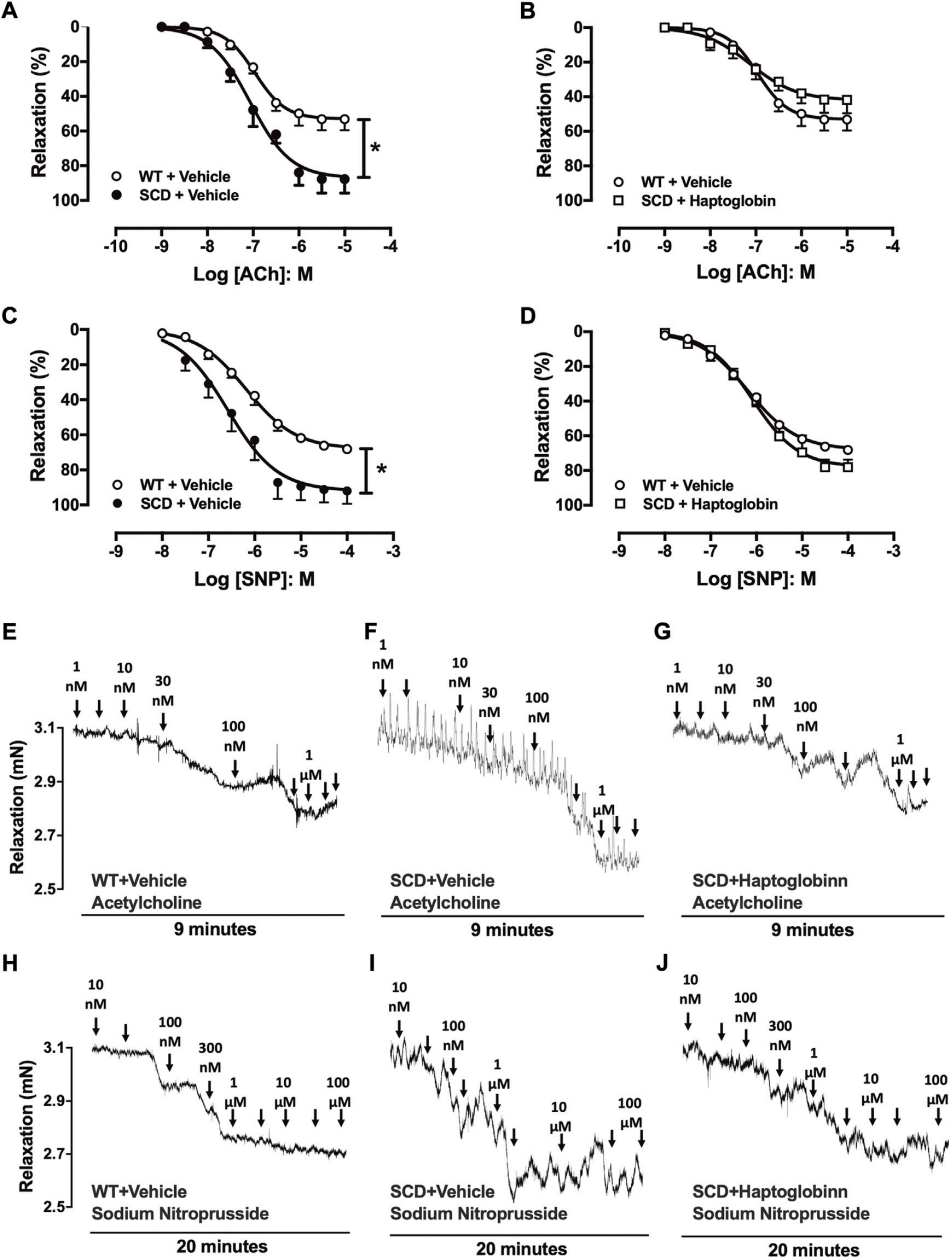
Concentration-response curves to acetylcholine [ACh; **(A,B)**] and sodium nitroprusside [SNP, **(C,D)**] in corpus cavernosum strips from WT and SCD mice treated or not with haptoglobin (Hap). Data were calculated relative to the maximal changes from the contraction produced by phenylephrine (10^–5^ M) in each tissue, which was taken as 100%. Data represent the mean ± SEM for 6 mice in each group. *p < 0.05 vs. WT-Vehicle; ^#^ *p* < 0.05 vs. SCD-Hap. Representative tracings of relaxant response to ACh **(E–G)** and SNP **(H–J)** in corpus cavernosum strips from WT and SCD mice treated or not with haptoglobin.

The cumulative addition of ACh (10^–9^ to 10^–5^ M) to PE-contracted tissues produced concentration-dependent relaxations in all groups (Figures 1A,B). However, maximal response (E_max_) produced by ACh was significantly higher in SCD compared with the WT group (*p* < 0.05; Figure 1A; Table 1). Haptoglobin treatment of SCD mice reduced the Emax values of ACh (*p* < 0.05; Table 1). No significant differences in potency (pEC_50_) for ACh was found among the groups. The pEC_50_ values for the ACh are shown in Table 1.

**TABLE 1:** Potency (pEC_50_) and maximal responses (E_max_) values obtained from concentration-response curves in cavernosal strips from WT and SCD mice treated with vehicle or haptoglobin.

	WT-Vehicle		SCD-Vehicle		SCD-Haptoglobin	
	pEC_50_	E_max_ (%)	pEC50	Emax (%)	pEC50	Emax (%)
ACh	6.96 ± 0.07	53 ± 6	7.06 ± 0.07	87 ± 7*	7.12 ± 0.14	42 ± 6#
SNP	6.18 ± 0.03	68 ± 1	6.57 ± 0.10*	92 ± 7*	6.08 ± 0.06#	78 ± 4

Concentration-response curves to acetylcholine and sodium nitroprusside in corpus cavernosum strips from WT, and SCD, mice treated with vehicle or haptoglobin. Data represent the mean ± S.E.M., of 6 experiments. *p < 0.05 compared with WT-Vehicle; #*p* < 0.05 compared with untreated SCD-Vehicle.

The cumulative addition of SNP (10^–8^ to 10^–4^ M) also produced concentration-dependent relaxations in all groups (Figures 1C,D). The E_max_ produced by SNP was significantly higher (*p* < 0.05) in CC from SCD compared to WT mice (Figure 1C; Table 1), which was reduced by treatment with haptoglobin (Figure 1D). SNP pEC_50_ value was significantly higher (*p* < 0.05) in the CC of SCD compared to WT mice (Figure 1C; Table 1), which was reversed (*p* < 0.05) by treatment with haptoglobin (Figure 1D; Table 1). Representative traces of responses to ACh and SNP are shown in Figures 1E–J.

**FIGURE 2:**
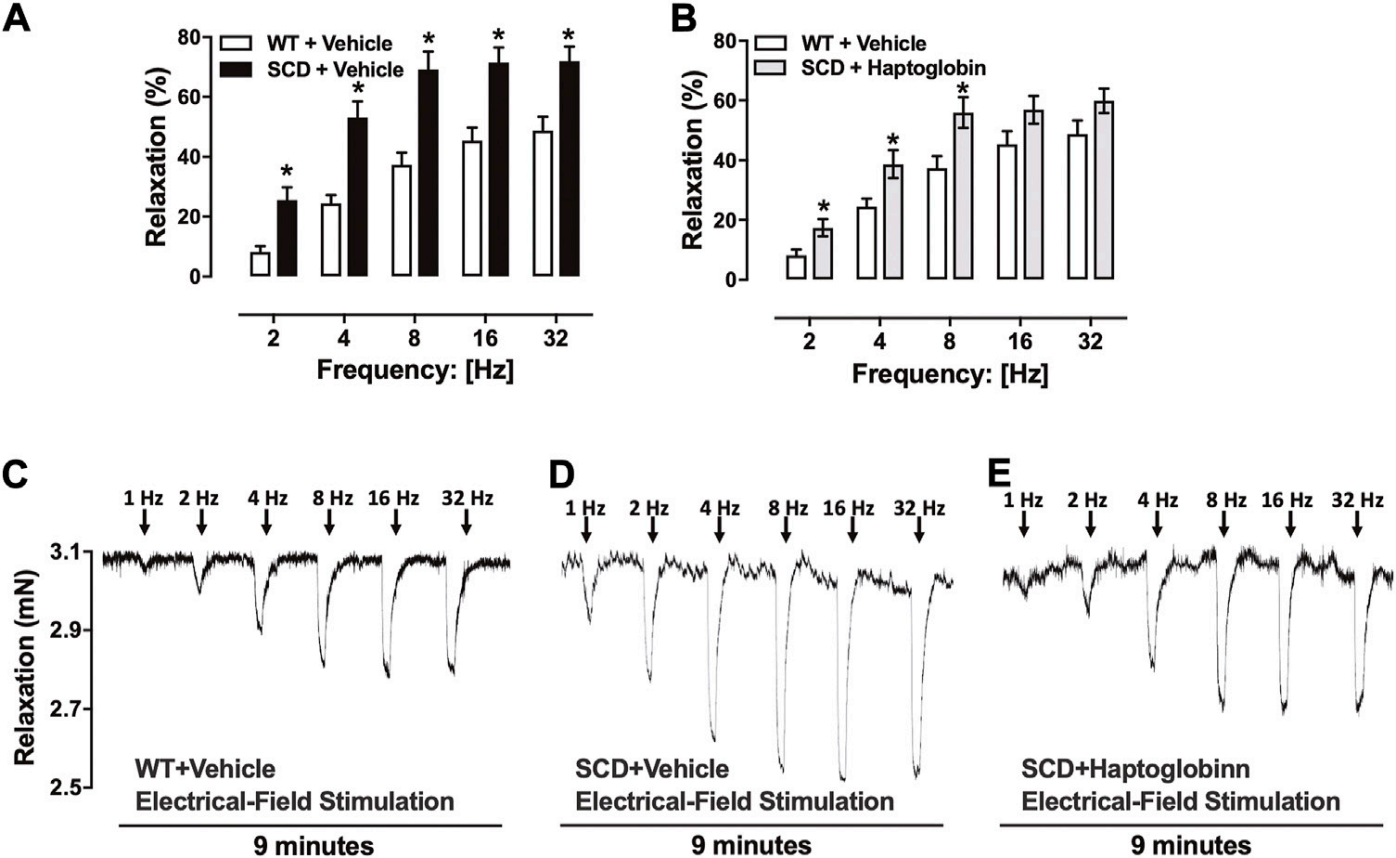
Relaxation responses induced by electrical-field stimulation (EFS) in corpus cavernosum strips WT **(A)** and SCD mice treated **(B)** or not with haptoglobin (Hap). Data were calculated relative to the maximal changes from the contraction produced by phenylephrine (10^–5^ M) in each tissue, which was taken as 100%. Data represent the mean ± SEM for 6 mice in each group. *p < 0.05 vs. WT-Vehicle. ^#^ *p* < 0.05 vs. SCD-Hap. Representative tracings of relaxant response to EFS in corpus cavernosum strips from WT **(C)** and SCD mice treated with vehicle **(D)** or haptoglobin **(E)**.

Electrical-field stimulation (EFS) of cavernosal tissues pretreated with guanethidine (3 × 10^–5^ M) and atropine (10^–6^ M) caused frequency-dependent mouse CC relaxations in all groups. CC relaxations to EFS were significantly higher (*p* < 0.05) in SCD compared to WT mice, as observed at 2–32 Hz (Figure 2A). Haptoglobin treatment reduced the EFS-induced increased relaxant responses in the CC from SCD (Figure 2B). Representative traces of responses to EFS are shown in Figures 2C–E.

### Haptoglobin treatment corrects reduced corpus cavernosum contractions in sickle cell disease mice

**FIGURE 3:**
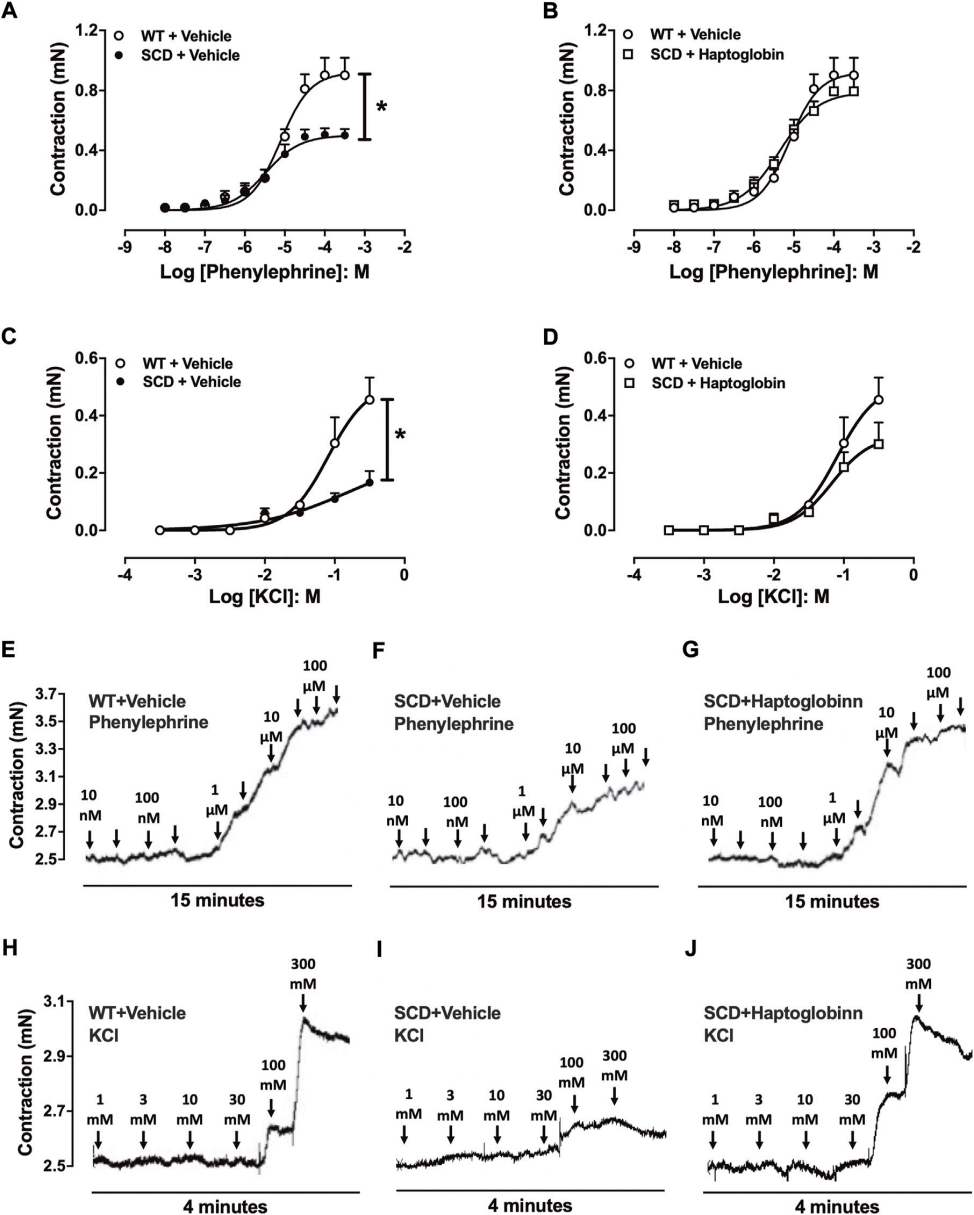
Concentration-response curves to phenylephrine **(A,B)** and KCl **(C,D)** in corpus cavernosum strips from WT and SCD mice treated or not with haptoglobin (Hap). Data are shown in mN, and data represent the mean ± SEM for 6 mice in each group. *p < 0.05 vs. WT-Vehicle. #*p* < 0.05 vs. SCD-Hap. Representative tracings of relaxant response to phenylephrine **(E–G)** and KCl **(H–J)** in corpus cavernosum strips from WT and SCD mice treated or not with haptoglobin.

Phenylephrine (10^–8^ to 3 × 10^–4^ M) induced concentration-dependent CC contractions in all groups (Figures 3A,B). The maximal response (Emax) was significantly lower (*p* < 0.05) in CC of SCD (0.50 ± 0.08 mN) compared to WT mice (0.90 ± 0.11 mn). Haptoglobin treatment of SCD mice increased the Emax values of phenylephrine in SCD group (0.79 ± 0.11 mn).

In the evaluation of receptor-independent stimulation, cumulative addition of KCl produced concentration-dependent CC contractions in all groups (Figures 3C,D). The maximal response (Emax) was significantly lower (*p* < 0.05) in CC of SCD mice (0.16 ± 0.04 mn) compared to WT mice (0.45 ± 0.08 mn). Haptoglobin treatment of SCD mice increased the Emax values of KCl in the SCD group (0.30 ± 0.07 mn). Representative traces of responses to phenylephrine and KCl are shown in Figures 3E–J.

### Haptoglobin treatment corrects downregulated p-eNOS (Ser-1177) and eNOS and does not affect nNOS protein expression in the sickle cell disease mouse penis.

**FIGURE 4:**
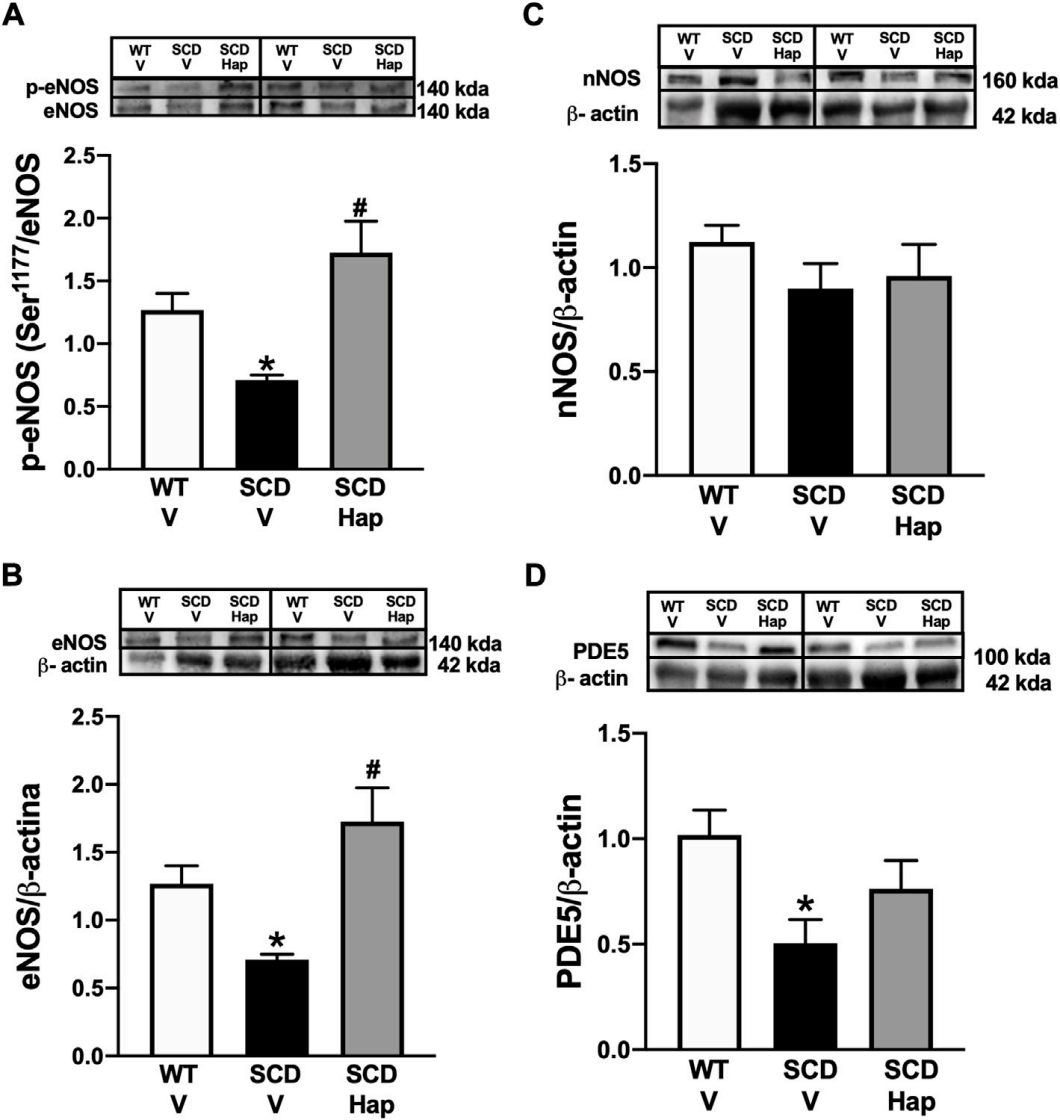
Representative images of western blotting (top panels) and protein values (bottom panels) for p-eNOS (Ser-1177; **(A)**, eNOS **(B)**, nNOS **(C)**, and PDE5 **(D)** in homogenates of penises from WT and SCD mice treated or not with haptoglobin (Hap). Data represent the mean ± S.E.M. for 6 mice in each group. A single band for eNOS, nNOS and PDE5 was normalized to β-actin. A single band for p-eNOS (Ser-1177) was normalized to eNOS. *p < 0.05 vs. WT-Vehicle. ^#^ *p* < 0.05 vs. SCD-Hap.

The protein expression for p-eNOS (Ser-1177) and eNOS was significantly reduced (*p* < 0.05) by approximately 45 and 44% in the penis of the SCD-vehicle group compared to the WT-vehicle group (Figures 4A,B, respectively). Haptoglobin treatment normalized (*p* < 0.05) the protein level of p-eNOS (Ser-1177) and eNOS in penis from SCD group. Protein expression of nNOS was not modified by SCD or haptoglobin treatment (Figure 4C).

### Haptoglobin treatment increased PDE5 protein expressions in the sickle cell disease mouse penis

The PDE5 protein expression was significantly (*p* < 0.05) decreased by approximately 50% in the penis of the SCD-vehicle group compared to the WT-vehicle group (Figure 4D). Haptoglobin treatment increased the protein level of PDE5 by approximately 52% in the penis of the SCD group (Figure 4D).

### Haptoglobin treatment restores ROCK2 and does not affect ROCK1 protein expressions in the sickle cell disease mouse penis.

**FIGURE 5:**
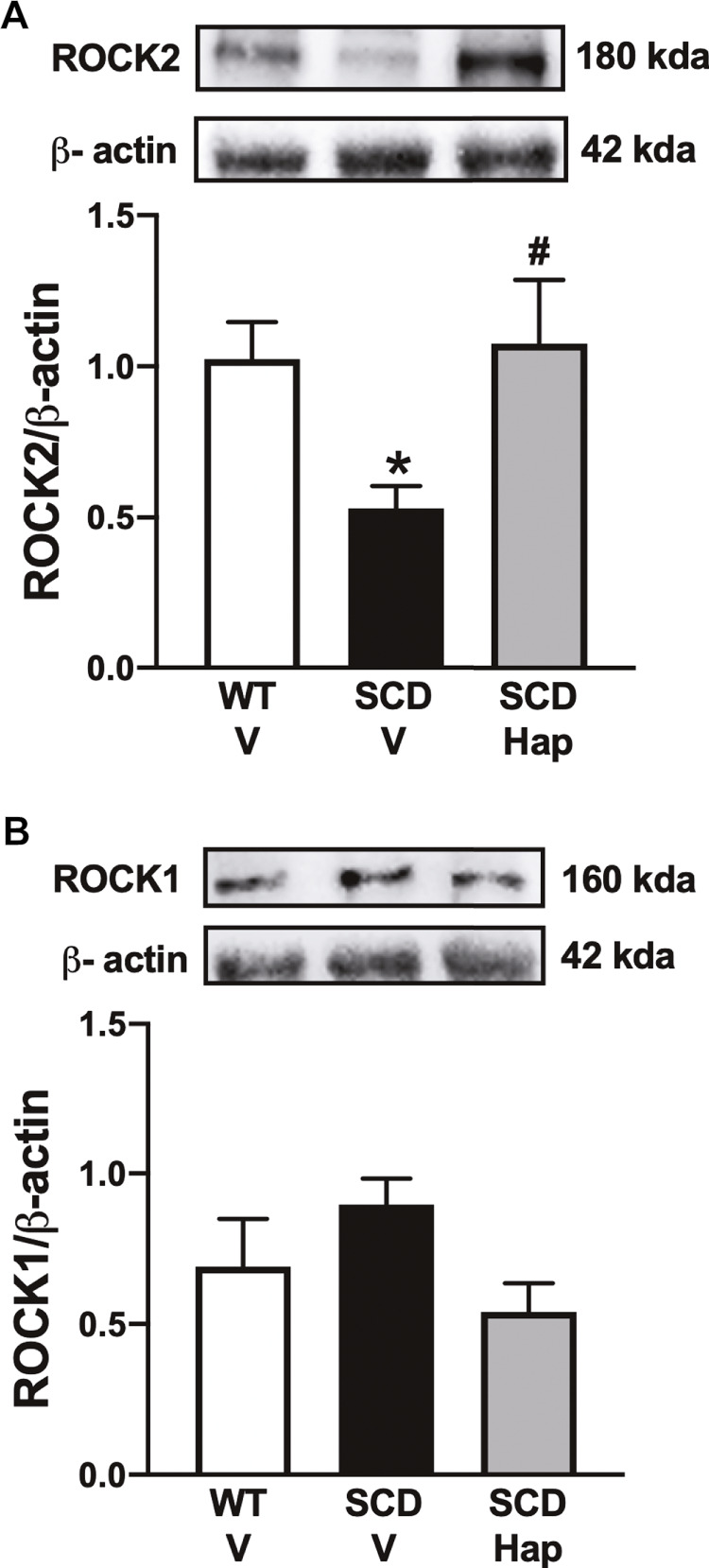
Representative images of western blotting (top panels) and protein values (bottom panels) for ROCK2 **(A)** and ROCK1 **(B)** in homogenates of penises from WT and SCD mice treated or not with haptoglobin (Hap). Data represent the mean ± S.E.M. for 6 mice in each group. A single band for ROCK1 and ROCK2 was normalized to β-actin. *p < 0.05 vs. WT-Vehicle; #*p* < 0.05 vs. SCD-Hap.

The protein expression for ROCK2 was significantly reduced (*p* < 0.05) by approximately 48% in the penis of the vehicle-treated SCD group in comparison with vehicle-treated WT mice (Figure 5A). Treatment with haptoglobin fully restored the protein levels of ROCK2 in the SCD group (Figure 5A). Protein expression of ROCK1 was not modified by SCD or haptoglobin treatment compared to the WT-vehicle group (Figure 5B).

### Haptoglobin treatment corrects increased oxidative stress and upregulated protein expression of NADPH oxidase subunit gp91phox in the sickle cell disease mouse penis.

**FIGURE 6:**
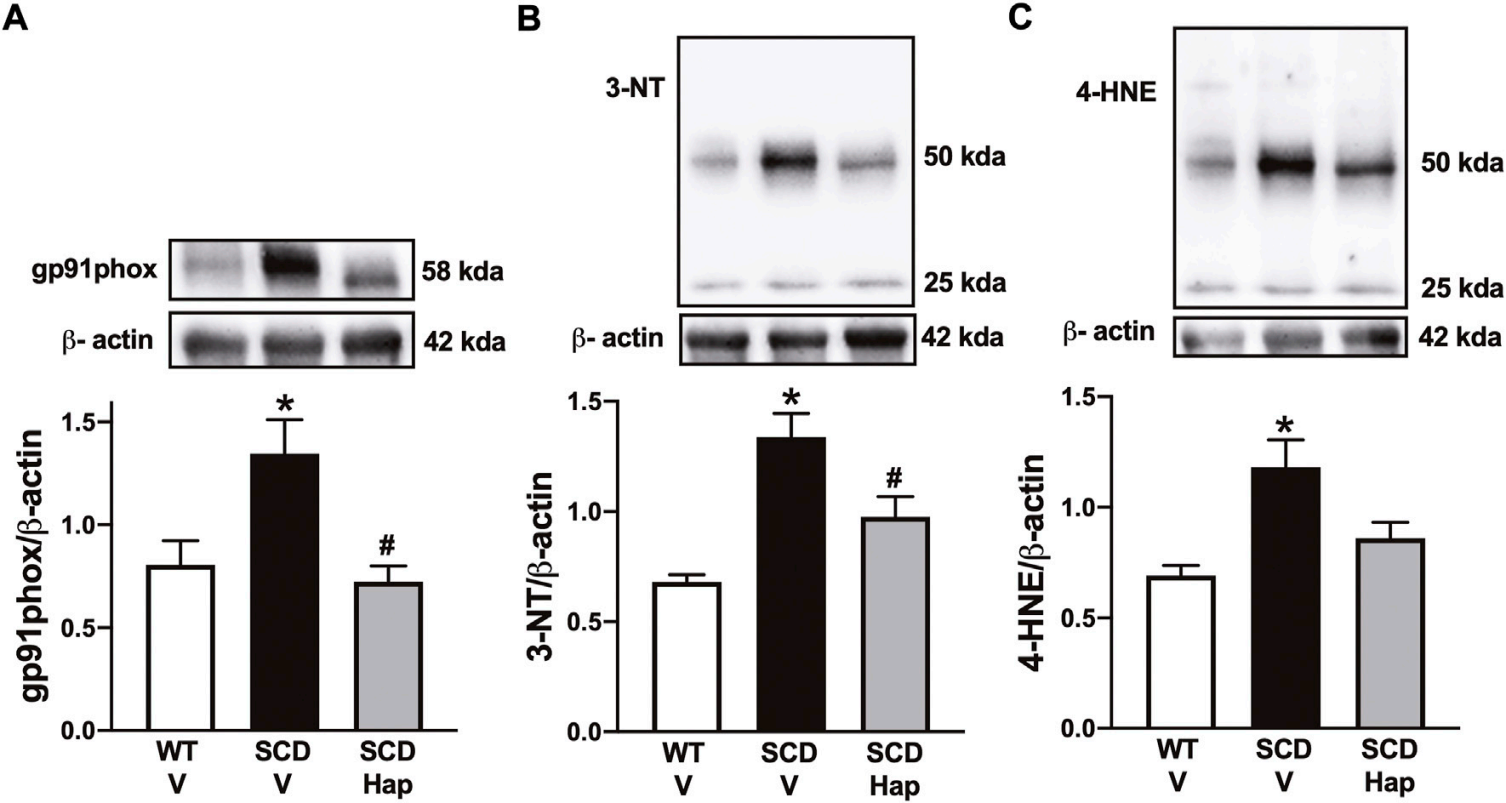
Representative images of western blotting (top panels) and protein values (bottom panels) for gp91^phox^ **(A)**, 3-NT **(B)**, and 4-HNE **(C)** in homogenates of penises from WT and SCD mice treated or not with haptoglobin. Data represent the mean ± S.E.M. for 6 mice in each group. A single band for gp91phox, 3-NT and 4-HNE was normalized to β-actin. *p < 0.05 vs. WT-Vehicle; ^#^ *p* < 0.05 vs. SCD-Hap.

The protein expression of gp91phox was significantly higher (*p* < 0.05) by approximately 67% in the penis of the vehicle-treated SCD group in comparison with vehicle-treated WT mice (Figure 6A). Treatment with haptoglobin fully normalized the protein levels of gp91phox in the SCD group (Figure 6A).

The protein expression for 3-nitrotyrosine and 4-HNE was significantly higher (*p* < 0.05) by approximately 95 and 75% in penile tissue from SCD in comparison with the WT group, respectively (Figures 6B,D). Haptoglobin treatment reduced the protein level of 3-nitrotyrosine and 4-HNE by approximately 27 and 28% in the SCD group (Figures 6B,D).

### Haptoglobin treatment increased cGMP levels in the sickle cell disease mouse penis

**FIGURE 7:**
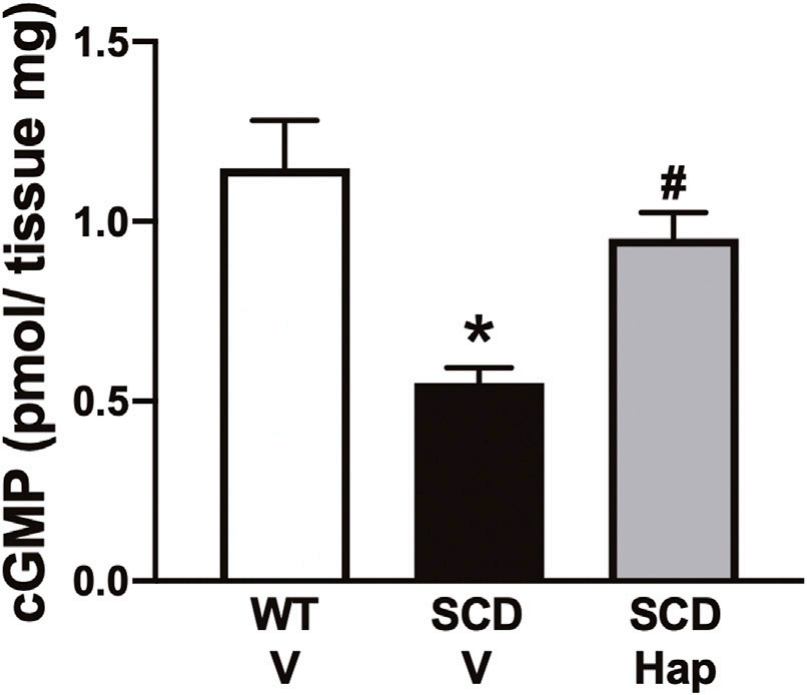
Basal GMP contents of penis from WT and SCD mice treated or not with haptoglobin. Data represent the mean ± S.E.M. for 6 mice in each group. *p < 0.05 vs. WT-Vehicle; ^#^ *p* < 0.05 vs. SCD-Hap.

The basal cGMP content in the erectile tissue was 60% lower (*p* < 0.05) in penises of SCD mice compared with WT-vehicle mice (Figure 7). Haptoglobin treatment increased (*p* < 0.05) the cGMP levels by approximately 72% in the penis of the SCD group (Figure 7).

## Discussion

The NO generated after erectile stimulation is the essential molecule that is produced in the penis to cause a penile erection. NO is produced by the endothelium that lines the sinusoids of the CC and also by nitrergic neurons (Hurt et al., 2012). ACh promotes the relaxation of the CC by stimulating the production of NO by the endothelium. In our study, the relaxation produced by ACh was greater in the CC of the SCD group. EFS promotes relaxation through neurogenic stimulation that results in NO production by nitrergic fibers (Andersson, 2011). The SNP is a NO-donor compound that is used as a pharmacological tool to assess endothelium-independent relaxation (Kowaluk et al., 1992). In our study, SNP-induced relaxation was also higher in the SCD group. Previous studies have reported that the increased cavernosal relaxation induced by cholinergic and nitrergic stimulation is due to the low expression of PDE5 in the penis (Silva et al., 2016b; Musicki et al., 2020). Therefore, when NO is released from the nitrergic fiber, endothelium or NO donors, diffuses into smooth muscle, and activates GCs, cGMP is produced, but PDE5 does not efficiently degrade it; thus, cGMP accumulates in the smooth muscle cell promoting CC relaxation and excess penile erection in SCD (Musicki and Burnett, 2020). Long-term treatment with haptoglobin reduced relaxation induced by ACh, EFS, and SNP in CC from the SCD group. The improvement in erectile function by haptoglobin treatment is likely due to the increases in PDE5 expression and decreased oxidative stress in the penises of SCD mice.

PDE5 expression is positively regulated by basal levels of cGMP in the penis (Lin et al., 2002). Penises from men and mice with SCD display lower basal production of NO due to lower expression and activity of eNOS (Bivalacqua et al., 2013; Lagoda et al., 2013; Silva et al., 2016a; Musicki et al., 2018). The lower bioavailability of NO results in reduced activation of GCs, a heterodimeric enzyme that contains a heme group that catalyzes the synthesis of the second messenger cGMP (Horst and Marletta, 2018). In our study, the expression of total eNOS and phosphorylated eNOS at its positive regulatory site Ser-1177 was normalized by haptoglobin treatment in the SCD group, whereas no change was observed in the expression of nNOS. These results indicate that basal NO production was increased in the penises of SCD mice. In fact, in our study, in accordance with previous studies (Champion et al., 2005; Bivalacqua et al., 2013), baseline cGMP levels were lower in the penises of the SCD group and haptoglobin treatment increased baseline cGMP levels in the SCD group, indicating an improvement in endothelial function. Previous studies have shown that intravascular hemolysis promotes endothelial dysfunction associated with reduced eNOS activity in SCD mice (Hsu et al., 2007), whereas haptoglobin preserves the NO signaling pathway in arteries during hemolysis (Schaer et al., 2016). Treatment with haptoglobin also increased PDE5 expression in the penises of SCD mice. Taken together, these results indicate that the improvement in endothelial function and increased cGMP levels may contribute to the increased expression of PDE5 in the penis of mice treated with haptoglobin. The increase in the expression of PDE5 in the smooth muscle of the CC prevented the excessive relaxation induced by cholinergic and nitrergic stimulation in SCD mice.

Noradrenaline released from the sympathetic nervous system induces contractions in the CC due to postjunctional activation of α1-adrenoceptors coupled to Gq protein that activates phospholipase C, which catalyzes the cleavage of phosphatidylinositol into inositol trisphosphate and diacylglycerol, thus increasing intracellular calcium levels (Mitidieri et al., 2020). Calcium produces smooth muscle contractions due to its binding to calmodulin, which activates myosin light chain kinase (MLC), leading to phosphorylation of MLC (Andersson, 2011). During this process, phosphorylated MLC interacts with beta-actin, resulting in smooth muscle contraction and maintenance of the penis in the flaccid state. (Andersson, 2011; Mitidieri et al., 2020). KCl promotes receptor-independent contraction, acts by depolarizing the smooth muscle cell membrane, and promotes calcium influx (Andersson, 2011). Our study is the first to show that contractions induced by the α1-adrenoceptor agonist and KCl were lower in the SCD mice. Activation of the RhoA/Rho-kinase signaling pathway increases Ca^2+^ sensitivity, thus participating in the contractile mechanism of CC smooth muscle. (Chitaley et al., 2001). There are two isoforms of ROCK, named ROCK1 and ROCK2 that share 65% homology (Nakagawa et al., 1996; Chitaley et al., 2001). However, the specific isoform functions of ROCKs are not well established, and their participation in erectile function alterations depends on the condition’s etiology (Sopko et al., 2014). Our study showed that ROCK2 expression is lower in CC of SCD mice, in agreement with a previous study (Bivalacqua et al., 2010). The reduction of ROCK2 is in agreement with our functional study, where contractions are reduced in SCD mice. Treatment with haptoglobin increased the contraction induced by phenylephrine and KCl and the expression of ROCK2. Mice deficient for the eNOS enzyme display reduced NO bioavailability associated with lower RhoA/Rho-kinase activity in the penis, as well as a priapism phenotype (Bivalacqua et al., 2007). In our study, haptoglobin treatment restored eNOS expression in the SCD group, indicating that normalization of NO bioavailability in the endothelium is associated with increased ROCK2 expression in the penis.

Elevated plasma hemoglobin and heme levels promote vascular and oxidative damage in SCD (Schaer et al., 2013; Rifkind et al., 2014; Chintagari et al., 2015). Experimental evidence shows that increased oxidative stress plays an essential role in the pathophysiology of priapism (Musicki et al., 2012, 2020; Lagoda et al., 2013; Silva et al., 2016b). Excess superoxide anion reacts with NO, forming peroxynitrite, a potent oxidizing agent (Pacher et al., 2007). In our study, haptoglobin treatment reduced gp91^phox^ expression, indicating a lower production of superoxide anion in the penis. According to this result, the expression of markers of oxidative stress (4-HNE) and nitrosative stress (3-NT) was reduced by haptoglobin treatment. The expression of gp91^phox^ is downregulated by a cGMP-dependent mechanism in the penis (Teixeira et al., 2007; Nunes et al., 2015), whereas exogenous NO inhibits NADPH oxidase activity through direct s-nitrosylation of the p47phox subunit in human endothelial cells (Selemidis et al., 2007). In SCD mice penises, treatment with molecules that increase the bioavailability of NO/cGMP reduced the expression of the gp91^phox^, 3-NT, and 4-HNE, as well as reversed priapism (Bivalacqua et al., 2013; Lagoda et al., 2014; Silva et al., 2016b; Musicki et al., 2018, 2020). Studies have reported that haptoglobin administration reduces vaso-occlusion and hemoglobin-mediated oxidative damage in SCD mice (Belcher et al., 2014, 2018; Chintagari et al., 2015). In our study, the normalization of gp91^phox^ expression by haptoglobin treatment may improve endothelial function.

We acknowledge several possible limitations of our study. First, although we focused on the effect of haptoglobin on the gp91^phox^ as it plays an important role in the pathophysiology of priapism, haptoglobin may have other effects on oxidative stress, such as reducing ROS sources; for example, all the isoforms of NADPH oxidase, unaccompanied eNOS, and xanthine oxidase. Moreover, haptoglobin may also affect antioxidant enzymes, such as glutathione peroxidase-1, which is reduced in the penises of sickled mice (Musicki et al., 2012). Second, although functional results with CC indicate that haptoglobin improves erectile function in sickle mice, detumescence time studies would further corroborate the relationship between molecular studies, functional studies, and the *in vivo* physiological response. Third, we did not treat WT mice with haptoglobin; additional studies are needed to assess the effect of haptoglobin on the physiology of erectile function.

In summary, the results from the present study suggest that long-term treatment with haptoglobin improved erectile function due to up-regulation of eNOS-PDE5 expression and down-regulation of the isoform of NADPH oxidase (gp91phox) and oxidative/nitrosative stress in the penises of SCD mice. Treatment with haptoglobin also increased contractile activity due to up-regulation of ROCK2. Our results support clinical studies that reported intravascular hemolysis contributes to the development of priapism in SCD. Therefore, haptoglobin treatment may be an additional strategy to prevent priapism in SCD.

## Data Availability

The raw data supporting the conclusions of this article will be made available by the authors, without undue reservation.
